# Supplementary material for: Hybrid Immunity from Gam-COVID-Vac Vaccination and Natural SARS-CoV-2 Infection Confers Broader Neutralizing Activity against Omicron Lineage VOCs Than Revaccination or Reinfection
Source: Vaccines (Basel). 2024 Jan 6;12(1):55. doi: 10.3390/vaccines12010055 (PMC10818410; doi:10.3390/vaccines12010055)
Supplement: Supplementary file 1 [file vaccines-12-00055-s001.zip › vaccines-2724485-supplementary.pdf]

Table S1 – Antibody titer analysis for Re-Vaccinated (RV) donors

№	IgM, CP	IgA, CP	IgG, BAU/ ml	Ncc, IgG, CP	Date of Vaccina-tion	Date of Re-Vaccina-tion	Date of Blood Sampling	Days between Vac. and Re-Vac.	Days before Blood Sam-pling
2	0.1	0.1	28.3	0.2	02.21	08.21	08.10.21	180	60
3	0.3	6.8	1023	0.2	21.04.21	27.10.21	19.11.21	185	21
4	0.2	4.5	1022	0.4	02.03.21	16.08.21	19.11.21	165	93
5	0.2	0.1	86	0.3	29.04.21	16.08.21	18.11.21	102	92
6	0.1	0.3	188.2	0.2	05.04.21	07.10.21	18.11.21	182	41
7	0.1	1.2	264.4	0.2	xx.12.20	xx.07.21	08.10.21	210	90
8	0.8	1.7	857	0.6	15.03.21	17.09.21	18.11.21	182	61
9	0.2	2.6	1022	0.3	26.03.21	09.2021	30.11.21	170	60
14	0.2	0.8	75	0.2	15.04.21	27.09.21	23.11.21	162	56
15	0.1	1.5	181	0.2	01.03.21	01.09.21	23.11.21	180	82
16	0.1	0.3	99	0.2	01.03.21	01.09.21	23.11.21	180	82
17	0.1	3.0	50	0.3	09.04.21	18.10.21	23.11.21	188	35
18	1.4	0.5	1696	0.2	03.03.21	06.09.21	23.11.21	183	77
19	0.1	1.1	440	0.2	01.02.21	06.08.21	23.11.21	185	105
21	0.2	0.5	2085	0.6	03.03.21	06.09.21	24.11.21	185	78

CP – coefficient of positivity; BAU – binding antibody units; Ncc – nucleocapsid protein of SARS-CoV-2

Table S2 – Antibody titer analysis for Re-Infected (RI) donors

№	IgM, CP	IgA, CP	IgG, BAU/ ml	Ncc, IgG, CP	Date of PCR1+ Patient's Condition	Date of PCR2+ Patient's Condition	Date of Blood Sampling	Days between PCR1+ and PCR2+	Days before Blood Sam-pling
23	0.6	1.7	441	16.8	21.06.21 Severe	13.10.21 Mild	23.11.21	114	40
24	0.0	5.1	409	5.9	07.10.20 Moderate	12.07.21 Moderate	23.11.21	280	131
25	17.7	2.5	470	17.6	22.10.20 Mild	25.10.21 Mild	25.11.21	368	30
26	0.1	5.3	1072	2.4	11.09.20 Moderate	- Mild	18.11.21	-	-
27	1.1	9.9	342	1.7	04.11.20 Moderate	- Mild	18.11.21	-	-
28	0.3	1.9	98	8.3	16.11.20 Mild	30.08.21 AS	18.11.21	290	79
30	0.6	9.6	263.1	17.7	20.06.20 Moderate	18.10.21 Moderate		430	?
33	0.4	4.0	753	3.0	02.2020 N/A	02.2021 N/A	18.11.21	360	280
34	0.4	8.7	112	1.3	04.03.21 Moderate	28.06.21 Mild	25.11.21	114	150
35	0.1	1.0	57.8	1.2	13.10.20 Moderate	21.06.21 Mild	16.11.21	250	147
36	0.5	4.9	486	4.6	01.10.20 Moderate	- AS	18.11.21	-	-
37	0.6	15.9	710	17.3	07.10.20 Moderate	12.07.21 AS	17.11.21	280	125
38	4.6	0.4	10	0.3	17.08.20 Mild	26.10.21 AS	23.11.21	430	27
82	7.4	16.8	2813	17.7	19.10.20 Moderate	29.10.21 AS	17.11.21	375	22

CP – coefficient of positivity; BAU – binding antibody units; Ncc – nucleocapsid protein of SARS-CoV-2; AS – asymptomatic; N/A – not available

Table S3 – Antibody titer analysis for Breaktrough Infected (BI) donors

№	IgM, CP	IgA, CP	IgG, BAU/ ml	Ncc, IgG, CP	Date of Vaccina-tion	Date of PCR+	Date of Blood Sampling	Days between Vac. and PCR+	Days before Blood Sam-pling
39	0.6	2.7	2123	17.7	20.07.21	13.09.21	18.11.21	53	65
40	0.7	11.7	4564	17.8	16.08.21	25.10.21	18.11.21	70	23
42	1.2	6.7	4220	4.8	15.05.21	11.10.21	25.11.21	68	45
43	0.6	0.8	202	0.8	09.06.21	30.06.21	23.11.21	140	143
44	0.4	8.9	475	13.8	10.06.21	05.07.21	18.11.21	25	132
45	2.0	15.2	4094	17.2	02.2021	xx.11. 21	-	-	-
46	0.7	11.1	4985	17.2	26.02.21	18.10.21	17.11.21	235	30
47	0.2	0.5	485	2.2	21.04.21	17.06.21	17.11.21	56	150
48	1.1	6.0	16498	17.7	05.05.21	04.10.21	17.11.21	150	43
49	0.3	6.5	9450	4.1	21.04.21	25.10.21	17.11.21	184	22
50	0.5	6.0	2498	17.3	26.07.21	24.09.21	24.11.21	58	60
51	0.2	0.9	368	1.9	19.05.21	02.08.21	23.11.21	73	111
52	0.2	17.3	1182	16.4	09.03.21	20.07.21	24.11.21	131	124
53	0.5	9.0	1413	17.3	21.07.21	20.09.21	24.11.21	59	64
54	1.1	18.4	4989	16.7	22.03.21	01.11.21	24.11.21	230	23
55	4.5	11.3	4695	17.2	13.05.21	03.11.21	24.11.21	170	21
56	0.3	2.2	969	7.2	04.06.21	31.07.21	24.11.21	113	112
57	0.1	2.8	1028	9.0	21.07.21	30.08.21	24.11.21	39	84
58	0.3	3.1	1675	15.5	01.05.21	01.10.21	24.11.21	150	54
59	3.1	18.4	50890	17.7	05.05.21	20.10.21	24.11.21	165	34
60	0.4	4.6	3085	17.7	05.05.21	01.10.21	24.11.21	145	54
61	0.4	1.2	1753	7.1	13.07.21	06.08.21	19.11.21	23	103
84	0.2	3.2	1231	5.9	21.04.21	07.07.21	17.11.21	76	130
85	0.2	11.2	772	1.6	11.06.21	02.08.21	24.11.21	51	112

CP – coefficient of positivity; BAU – binding antibody units; Ncc – nucleocapsid protein of SARS-CoV-2

Table S4 – Antibody titer analysis for Vaccinated Convalescent (VC) donors

№	IgM, CP	IgA, CP	IgG, BAU/ ml	Ncc, IgG, CP	Date of PCR+	Date of Vaccina-tion	Date of Blood Sampling	Days between PCR+ and Vac.	Days before Blood Sam-pling
62	1.9	0.6	1418	13.3	16.09.20	16.08.21	18.11.21	330	92
63	0.3	4.1	8608	13.7	16.11.20	27.09.21	18.11.21	314	51
64	3.2	18.0	14129	14.6	10.11.20	06.09.21	18.11.21	296	72
65	0.6	16.9	2850	3.3	26.10.20	16.08.21	18.11.21	300	92
67	0.1	6.1	4434	1.7	21.10.20	21.09.20	18.11.21	330	57
68	1.1	16.6	4569	1.7	14.05.20	xx.07.21	10.09.21	420	60
69	1.0	10.4	3042	3.0	30.10.20	16.08.21	18.11.21	286	92
70	0.6	9.2	474	1.1	04.11.20, 30.06.21	17.07.21	18.11.21	253	121
72	0.7	7.8	1190	5.9	xx.10.20	xx.06.21	17.11.21	240	150
83	1.3	1.7	827	3.9	26.10.20	17.07.21	18.11.21	251	121
75	0.4	12.0	733	0.7	11.09.20	16.08.21	18.11.21	335	92
76	1.3	5.0	731	0.4	10.09.20	16.08.21	18.11.21	336	92
80	0.4	8.9	2657	13.7	17.06.21	18.10.21	24.11.21	121	35
81	0.5	3.8	1087	0.8	18.11.20	06.09.21	24.11.21	288	78

CP – coefficient of positivity; BAU – binding antibody units; Ncc – nucleocapsid protein of SARS-CoV-2

Table S5 – Neutralization of virus variants by Re-Vaccinated (RV) donor sera (GMT ID50)

№	Wu-1	BA.1	BA.4/5
2	11	10	10
3	396	84	54
4	864	77	107
5	50	10	10
6	146	11	10
7	17	10	10
8	807	43	10
9	1582	84	33
14	53	10	10
15	42	10	10
16	105	10	10
17	232	168	15
18	899	122	37
19	387	52	13
21	2058	159	41

Table S6 – Neutralization of virus variants by Re-Infected (RI) donor sera (GMT ID50)

№	Wu-1	BA.1	BA.4/5
23	205	24	24
24	515	74	116
25	901	9	16
26	154	46	19
27	247	45	40
28	94	38	26
30	535	35	111
33	338	95	11
34	85	9	10
35	115	52	68
36	928	72	71
37	1936	15	76
82	2135	76	236

Table S7 – Neutralization of virus variants by Breakthrough Infected (BI) donor sera (GMT ID50)

№	Wu-1	BA1	BA.4/5
39	15398	107	96
40	5770	169	167
42	4531	280	671
43	53	10	10
44	209	44	33
45	3138	246	670
46	3161	320	562
47	658	61	37
48	10536	352	1440
49	8165	968	744
50	1346	126	152
53	1469	129	30
54	6834	1106	542
55	4079	589	690
56	875	242	61
57	1163	22	28
58	3476	174	288
59	72083	27370	32159
60	2215	504	172
61	776	55	10
84	44172	ND	1751
85	10380	45	206

Table S8 – Neutralization of virus variants by Vaccinated Convalescents (VC) donor sera (GMT ID50)

№	Wu-1	BA.1	BA.4/5
62	1704	195	55
63	3915	2801	1679
64	6439	2120	1282
65	982	832	232
67	1351	1007	610
68	2231	699	541
69	1420	498	638
70	1917	488	143
72	10267	431	396
83	4405	66	57
75	770	279	108
76	1278	113	110
80	1731	286	109
81	14541	67	30
